# Happiness is Greater in More Scenic Locations

**DOI:** 10.1038/s41598-019-40854-6

**Published:** 2019-03-14

**Authors:** Chanuki Illushka Seresinhe, Tobias Preis, George MacKerron, Helen Susannah Moat

**Affiliations:** 1 Data Science Lab, Behavioural Science, Warwick Business School, University of Warwick, Coventry, CV4 7AL UK; 2 The Alan Turing Institute, British Library, 96 Euston Road, London, NW1 2DB UK; 3 Department of Physics, Boston University, 590 Commonwealth Avenue, Boston, Massachusetts 02215 USA; 4 Department of Economics, University of Sussex, Jubilee Building, Falmer, Brighton, BN1 9SL UK

## Abstract

Does spending time in beautiful settings boost people’s happiness? The answer to this question has long remained elusive due to a paucity of large-scale data on environmental aesthetics and individual happiness. Here, we draw on two novel datasets: first, individual happiness data from the smartphone app, *Mappiness*, and second, crowdsourced ratings of the “scenicness” of photographs taken across England from the online game *Scenic-Or-Not*. We find that individuals are happier in more scenic locations, even when we account for a range of factors such as the activity the individual was engaged in at the time, weather conditions and the income of local inhabitants. Crucially, this relationship holds not only in natural environments, but in built-up areas too, even after controlling for the presence of green space. Our results provide evidence that the aesthetics of the environments that policymakers choose to build or demolish may have consequences for our everyday wellbeing.

## Introduction

Areas of great natural beauty have long been considered to be locations in which one might hope to feel a greater sense of happiness. What characteristics of such environments might be driving such an effect? Is it simply the overwhelming presence of nature, or might the beauty of these environments be crucial? If aesthetics play a key role, might this apply in built-up environments too, where policy makers, urban planners, property developers, and architects can affect the design of the places we experience, and potentially therefore our everyday happiness?

The relationship between the environment and subjective wellbeing has been the subject of an extensive scientific literature^1–5^ as well as parliamentary briefings^6^. Experimental and survey based studies have produced a sequence of results suggesting that natural habitats are associated with greater happiness, a result usually explained with reference to the *‘biophilia hypothesis’*, which suggests that evolutionary pressures have led to a human preference for a connection with nature^7^. However, to date, researchers in this domain have had to contend with considerable limitations in measuring happiness levels as humans experience different environments^8^ as well as in measuring the aesthetics of those different environments.

Limitations in measuring subjective wellbeing have largely been due to the resources required to administer a survey to establish how happy an individual is. In experimental situations, this constraint has normally resulted in only one or two measurements being taken, for a restricted number of participants^1,2,9^. Where major survey initiatives have facilitated the collection of subjective wellbeing data for thousands of participants, questionnaires have usually been administered at most once a year^4,5^. Such approaches have not enabled researchers to measure the fluctuations in happiness that may occur as individuals experience a range of environments during their everyday life.

Similarly, researchers have had limited access to large-scale data on the beauty of the environment. In experimental settings where researchers have directly exposed participants to different environments, environments have been classified as either urban or natural^1,2^. In survey based studies of large numbers of participants, researchers have been able to draw on national scale data on the environment derived from remote imaging, such as data on whether an area is natural or built-up^3^, or how much green space is present in the local environment^4,5^. A new line of studies has asked participants to gather photographs of the environments they experience^10^. However, data on the aesthetics of the environments experienced by the participants have not been available for analysis.

Intriguingly, results from studies in which participants viewed sequences of images provide initial indications that photographs of environments considered more attractive are associated with improved mood^11–13^. An association has also been reported between satisfaction with the view from a workplace window and general wellbeing^14^. However, other than in the study reported by Pretty *et al*.^12^, who split a set of 30 photographs into pleasant and unpleasant scenes on the basis of feedback from a panel of 50 people, photos or views in these studies were rated for attractiveness by the same person reporting their mood or wellbeing, such that aesthetic and emotional responses to an image may be difficult to disentangle. Furthermore, while showing photographs to participants in a laboratory setting has provided the key previous opportunity to gain insight into the aesthetics of the scene viewed, it could also be argued that emotional reactions to environmental scenes in everyday life may differ. Some evidence of a link between viewing art and improved mood has also been reported, for example in patients with Alzheimer’s disease^15^.

**Figure 1:**
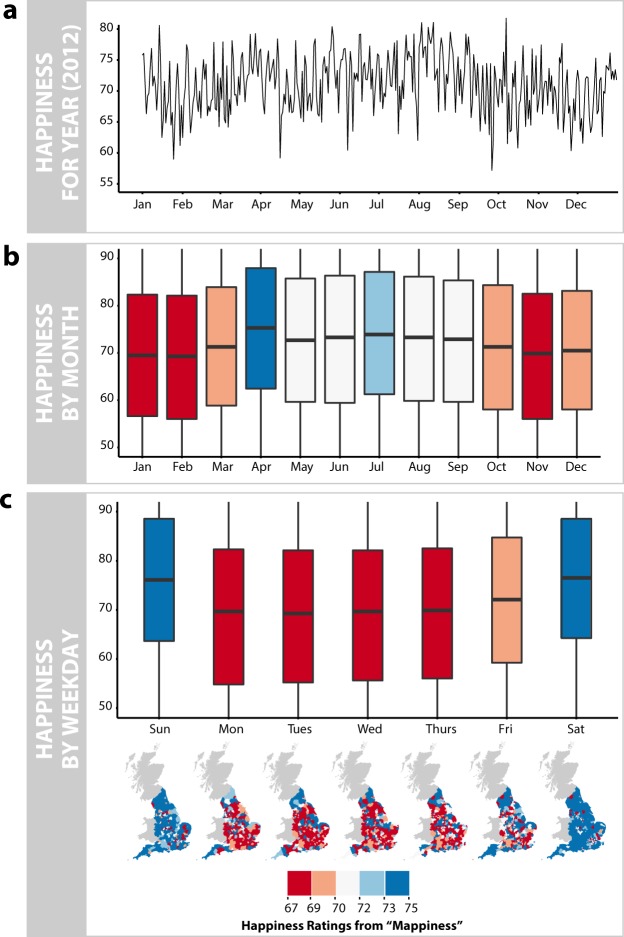
Measuring Happiness with Data from the Apple iOS App *Mappiness*. (**a**) We measure changes in individual happiness using data from *Mappiness*, an Apple iOS app that polls participants at random times throughout the day to ask them to report their wellbeing, as well as other details of their current situation, such as who they are with and what activities they are taking part in. Here we show how happiness varies over the year 2012. Trends and oscillations in the measurements suggest that happiness may vary depending on factors such as the month or day of the week. (**b**) We aggregate happiness ratings for all months. Visual inspection suggests that people tend to be less happy during the winter months. (**c**) Aggregation of happiness ratings by the day of the week shows that people are happiest at the weekends. Location data from *Mappiness* also allow us to visualise how happiness ratings might vary geographically. Across all parts of the figure, colour coding is based on breaks of equal intervals of aggregated weekly happiness ratings. We also draw the reader’s attention to the size of the variation associated with these factors, which given the full *Mappiness* scale of 0–100 is relatively small. The coefficients in Table 1 show that this is the case for all factors considered in this analysis.

Could the aesthetics of an environment therefore have a crucial association with everyday happiness that studies to date have not been able to capture? Recent methodological advances drawing on data from mobile phones and Internet activity have opened up new avenues for measuring human behaviour and experience which may allow us to provide an answer to this longstanding question^16,17^. Two recent studies are of particular relevance. First, MacKerron and Mourato^3^ present *Mappiness*, an Apple iOS smartphone app which allows users throughout the UK to track their happiness. The *Mappiness* app builds on the Experience Sampling Method (ESM), where participants are asked to use a diary to record details on their wellbeing and current situation at prespecified times of the day^18,19^. Figure 1 depicts how happiness ratings from the *Mappiness* app vary over time. The use of a smartphone app to poll participants allows MacKerron and Mourato^3^ to scale this methodology to tens of thousands of participants as it reduces the prohibitively high burden of the original diary-based method^20^. Moment-by-moment records might also provide a less distorted picture of an individual’s experiences^18,20^, as we do not have to rely on people’s recollection of their experiences, which are often susceptible to biases^21^. Such biases include the peak-end rule and duration neglect^22^. Crucially, the smartphone app is also able to automatically record the GPS location of a participant when they respond to the survey^10,23^. Second, Seresinhe, Preis and Moat analyse data from *Scenic-Or-Not*^24^, an online game in which players rate geotagged photographs taken across the United Kingdom, on the basis of how scenic they find them to be. Through this game, over 1.5 million ratings for photos of over 200,000 locations in the United Kingdom have been collected, producing national scale measurements of environmental aesthetics of a kind not previously available to researchers.

Here, we combine these two novel datasets, to exploit a unique opportunity to gather large-scale quantitative insight into whether individuals encountering more scenic environments during their everyday life experience greater levels of happiness. Does such a relationship hold even in built-up environments, rather than natural habitats, when taking other environmental measures such as green space into account? The answer to this question could have implications not only for theories of how the environment impacts upon our everyday wellbeing, but also for social and environmental policy, including whether the aesthetics of the built as well as natural environment warrant investment.

## Results

We use individual reports of momentary happiness from *Mappiness* in order to better understand how scenic areas might affect people’s wellbeing. In the *Mappiness* app, participants are asked to report their wellbeing at random times throughout the day. At time of polling, participants are also asked whether they are alone or with someone else, where they are (such as home, work, indoors or outdoors) and what activities they are taking part in.

We measure scenicness using crowdsourced scenic ratings from *Scenic-Or-Not* (http://scenicornot.datasciencelab.co.uk/). Participants rate *Scenic-Or-Not* photographs, each representing one square kilometre of Great Britain, on an integer scale of 1–10, where 10 indicates “very scenic” and 1 indicates “not scenic” (see Supplementary Fig. 1 for an image of the voting screen). We use the mean rating of images that have been rated at least three times. We aggregate the scenicness data to the level of Lower Layer Super Output Area (LSOA). LSOAs are areas defined by the UK’s Office of National Statistics for statistical analyses and have an average population size of 1,600 and an area spanning anywhere between 0.018 square km to 684 square km. The scenic rating of larger LSOAs will therefore be determined from a larger selection of images than the scenic rating of smaller LSOAs. We choose the level of LSOA for our analysis as this is the smallest area for which we have data on other environmental factors which we wish to take into account, such as percentage of green space and whether an area is urban, suburban or rural.

In order to determine whether individuals are happier in more scenic environments, we use fixed-effects (within) estimators, which are often applied in economic analyses exploring the connection between changes in environmental factors and wellbeing^3,5,25^. We choose this approach as fixed effects models help us to capture unobservable factors relating to an individual that are difficult to measure and do not change across time, such as personality traits, which may correlate both with our outcome variable, happiness, and the other explanatory variables in our model.

People may visit scenic environments with family or friends, when the weather is particularly good, when deciding to take a break in the rolling countryside, or simply for some exercise. As all these factors themselves might contribute to people’s happiness, we include a variety of control variables in our model, specifically: companionship, activities (such as walking, sports or gardening) and weather conditions. We also consider the time of day, separately for Monday to Friday or weekends and bank holidays. In addition, we consider whether a participant is at home, at work, or elsewhere when they respond to the *Mappiness* app. While the environmental qualities associated with being at home will stay fairly constant for many participants, this allows us to distinguish between situations in which participants are at home, and situations in which participants are in an environment with similar characteristics to where they live, but are not at home. To account for the fact that usage of the *Mappiness* app may itself affect happiness levels, we control for the number of previous responses by the same participant. We note that *Mappiness* measurements drawn from the same individual or same LSOA are unlikely to be independent. In order to ensure that such dependencies are accounted for in our statistical analysis, we cluster our standard errors on both the individual and LSOA level. More details on this model can be found in the *Methods* section.

Table 1 presents the results of our analysis. We note that all predictor variables take values in the range 0 to 1, aiding comparison of the coefficients from our regression analysis. Visual inspection of this table reveals that the directions of the relationships between many of the control variables and happiness are in line with what we might intuitively expect and accord with previous research. For example, commuting is negatively associated with happiness^26^ while leisure activities such as resting, gardening^27^, walking^28^ and spending time with family and friends^29^ are positively associated with happiness. Rain is associated with reduced happiness, while higher temperatures and more sunshine are associated with increased happiness^30^. Crucially however, we find that people do report themselves to be happier when in a more scenic location ($\beta =3.527$, $CI=[2.551,4.504]$, $p < 0.001$, $N=138\,407$), even after controlling for weather, activities, companionship, weekdays or weekends, and previous usage of the *Mappiness* app.

**Table 1:** Is Happiness Greater in More Scenic Locations? Estimated Model Parameters For Fixed Effects Model.

*Environment Variables*	Model 1: scenicness only		Model 2: scenicness and environmental variables	
	*Coeff*	*95*% *C*.*I*.	*Coeff*	*95*% *C*.*I*.
Scenicness	3.527***	[2.551, 4.504]	2.770***	[1.757, 3.783]
Natural habitat	—		0.574***	[0.303, 0.844]
Percentage of green space	—		−0.451	[−0.999, 0.0979]
Log of area-level median household income	—		−0.255	[−0.654, 0.144]
Urban	—		−0.282	[−0.668, 0.103]
Rural	—		0.608***	[0.263, 0.954]
Suburban (base category)	—		—	
*Participant is*…				
Home	0.375	[−0.113, 0.862]	0.442	[−0.0452, 0.930]
Work	−3.252***	[−3.764, −2.739]	−3.217***	[−3.730, −2.705]
Elsewhere (base category)	—		—	
*Companionship*				
Spouse, partner, girl/boyfriend	4.215***	[3.858, 4.572]	4.144***	[3.787,4.501]
Children	0.564*	[0.0622, 1.066]	0.556*	[0.0543,1.058]
Other family members	1.278***	[0.897, 1.659]	1.196***	[0.812,1.580]
Colleagues, classmates	0.0327	[−0.804, 0.869]	0.00123	[−0.833,0.835]
Clients, customers	2.593***	[1.311, 3.876]	2.566***	[1.280,3.853]
Friends	4.500***	[4.155, 4.846]	4.441***	[4.092,4.790]
Other people participant knows	−1.486***	[−2.147, −0.826]	−1.531***	[−2.192, −0.869]
*Selected Activities*				
Travelling, commuting	−2.216***	[−2.517, −1.914]	−2.214***	[−2.517, −1.911]
Sleeping, resting, relaxing	1.204***	[0.563, 1.845]	1.133***	[0.494, 1.773]
Talking, chatting, socialising	4.202***	[3.853, 4.552]	4.193***	[3.844, 4.542]
Eating, snacking	1.413***	[1.009, 1.816]	1.426***	[1.022, 1.829]
Walking, hiking	3.918***	[3.513, 4.324]	3.857***	[3.453, 4.261]
Sports, running, exercise	7.221***	[6.530, 7.913]	7.186***	[6.495, 7.878]
Gardening, allotment	3.955***	[3.103, 4.807]	3.958***	[3.105, 4.811]
Birdwatching, nature watching	4.143***	[3.233, 5.053]	3.979***	[3.064, 4.893]
Hunting, fishing	4.994***	[2.275, 7.713]	4.755***	[2.051, 7.460]
+33 further activity dummies	Yes		Yes	
*Weather*				
Wind speed	−1.337**	[−2.260, −0.414]	−1.362**	[−2.285, −0.440]
Cloud cover	−0.761***	[−1.164, −0.358]	−0.791***	[−1.195, −0.388]
Visibility	0.223	[−0.419,0.865]	0.186	[−0.456,0.828]
Temperature	4.018***	[2.745,5.292]	4.067***	[2.792,5.343]
Sun	1.149***	[0.772,1.525]	1.124***	[0.748,1.501]
Rain	−11.06***	[−16.69, −5.444]	−11.07***	[−16.69, −5.447]
Hours of weekday/weekend and bank holiday dummies (3-hour blocks)	Yes		Yes	
Mappiness usage dummies (participant’s response, 1, 2–11, 12–51)	Yes		Yes	
Observations	138,407		138,407	
Groups (participants)	15,444		15,444	
Groups (LSOAs)	14,228		14,228	
R^2^	49.5%		49.5%	
Adjusted R^2^	43.1%		43.1%	
Within R^2^	11.6%		11.6%	

*$p < 0.05$, **$p < 0.01$, ***$p < 0.001$. The dependent variable is Happiness, scaled to 0–100. Note that while all the activities that people report on in the *Mappiness* app have been included in the model (Supplementary Table 2), we only report the activities that we expect to be common in scenic environments. We find that people are happier when in more scenic locations, even after accounting for environmental factors such as presence of green space, or whether the location is a built-up area or a natural habitat.

### Comparing scenic environments to natural, green and rural environments

However, this analysis alone is not enough to allow us to determine whether the aesthetics of an environment play a role which goes beyond the role of nature that previous studies have considered. Indeed, intuitively we may understand scenic environments to be akin to natural environments or green spaces, with our preferences driven by an affinity for natural areas^7,31–33^. Similarly, it seems reasonable to suggest that the most scenic areas of the country may be rural areas rather than urban areas. We explore to what extent scenicness differs from these environmental factors.

**Figure 2:**
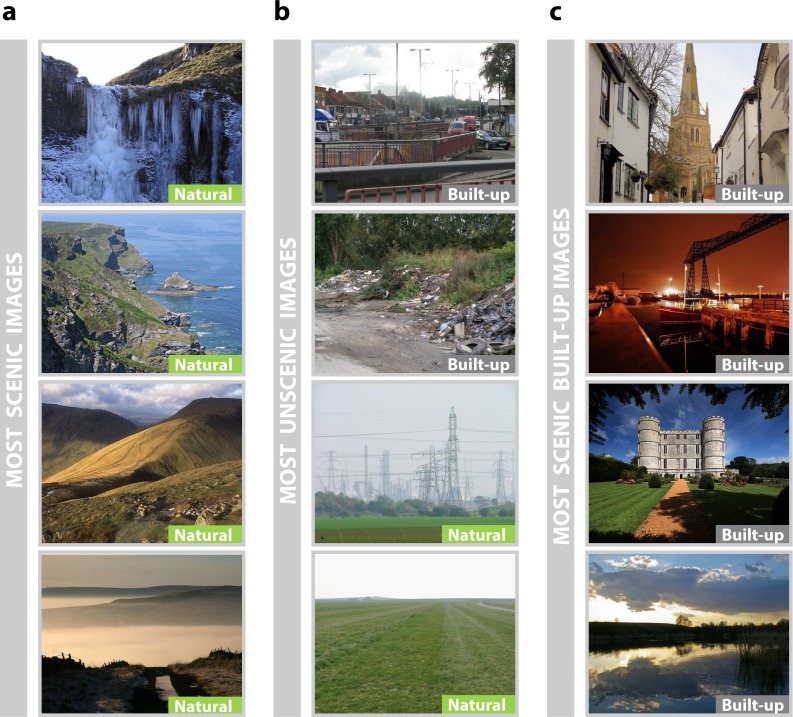
Scenic and Unscenic Images from *Scenic-Or-Not*. (**a**) The four most scenic images in England. Visual inspection suggests that scenic images are primarily composed of natural landscapes. They not only contain large areas of green space, but also mountainous landscapes and water features. (**b**) A sample of the most unscenic images. Such images tend to be taken in built-up areas and might include dense road networks or abandoned rubbish. However, natural areas can also be rated as highly unscenic if industrial structures obstruct the naturally scenic view or if they appear to be largely featureless or desolate. (**c**) A sample of the top 5% scenic images in built-up locations. Scenic images in built-up locations can include a variety of features such as quaint villages, industrial structures such as bridges, castle-like structures, and park lakes. Photographs shown in this figure have been cropped to fit. Photo credits: (**a**) From top to bottom: ⓒ Richard Swales, ⓒ Tony Atkin, ⓒ Tom Richardson, ⓒ Helen Wilkinson; (**b**) From top to bottom: ⓒ Peter Whatley, ⓒ David Long, ⓒ Mick Garratt, ⓒ Doug Lee; (**c**) From top to bottom: ⓒ Bob Jones, ⓒ Phil D, ⓒ Mike Searle, ⓒ Glyn. Copyright of the images is retained by the photographers. Images are licensed for reuse under the Creative Commons Attribution-Share Alike 2.0 Generic License. To view a copy of this licence, visit http://creativecommons.org/licenses/by-sa/2.0/. The full photographs and URLs are provided in the Supplementary Information.

In order to determine how scenicness ratings compare to classifications of environments as natural or built-up, we use land cover data^34^ to categorise the geo-located coordinates of each image for which we have a scenic rating as either a natural environment or a built-up environment. We find that the scenic ratings in natural environments (mean = 4.16, median = 4.14) do tend to be higher than the scenic ratings in built-up environments (mean = 2.86, median = 2.60; $W=326\,330\,000$, $p < 0.001$, $N=119\,377$, Wilcoxon rank sum test with continuity correction). Similarly, using data from the *2011 Rural-Urban Classification*^35^, we find that scenicness is greater in rural environments (mean = 4.19, median = 4.14) than in urban and suburban environments combined (mean = 3.33, median = 3.20; $W=703\,750\,000$, $p < 0.001$, $N=119\,377$, Wilcoxon rank sum test with continuity correction). However, Fig. 2 illustrates that images with low scenic ratings are not always taken in built-up environments, such that the distributions of scenic ratings in natural and in built-up environments do overlap (Supplementary Fig. 2).

We next compare scenicness to green space using the data on the percentage of green land cover per LSOA^36^, in line with a previous analysis of *Scenic-Or-Not* data and relationships to green space^24^. We find that scenicness is correlated with the percentage of green space, but the effect size is not very large (${\rho }_{\tau }=0.20$, $p < 0.001$, $N=119\,375$, Kendall’s rank correlation).

**Figure 3:**
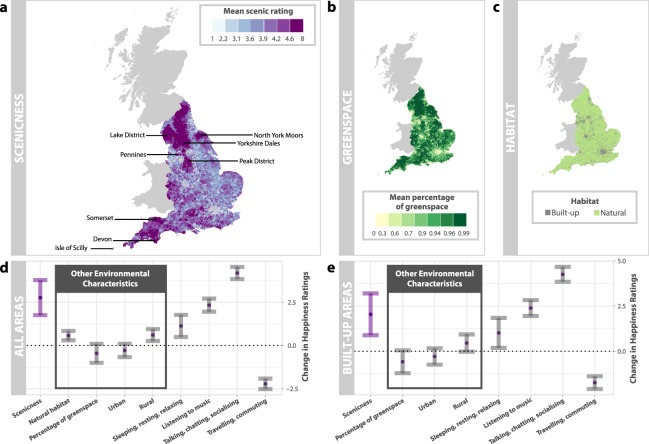
Happiness is Greater in More Scenic Settings. (**a**) We calculate the mean scenic rating of all *Scenic-Or-Not* photographs taken for each LSOA and depict these ratings using quantile breaks. Popular notions of scenic areas such as the Lake District and the Peak District are clearly visible on the map. (**b**) In order to understand whether scenic environments are simply green or natural environments, we consider data on the percentage of green land cover per LSOA^36^, depicted here using quantile breaks. (**c**) We also consider data on land cover types^34^, which we use to classify locations as natural environments or built-up environments. We find that scenic ratings are not equivalent to measurements of green space and are not entirely determined by whether an image was taken in a natural or built-up environment (see main text for analysis). (**d**) Coefficients of selected predictor variables and their 95% confidence intervals, based on results of a fixed effects model (for full results, see Table 1). The dependent variable is happiness, scaled to 0–100, and the coefficient size reflects the change in happiness rating associated with a change of one unit in the given predictor variable. We find that people are happier in more scenic environments, even when taking other traditional measurements of the environment into account. We observe that the predicted increase in happiness when moving from a location with the lowest possible scenic rating to a location with the highest possible scenic rating is similar in size to the increase in happiness predicted when participants are listening to music. (**e**) We find that this effect holds even within built-up areas, where policymakers have the ability to influence the aesthetics of the environments we live in. LCM2007 NERC (CEH) 2011. © Crown 2007, Ordnance Survey License number 100017572 third party licensors.

Finally, we investigate the extent to which natural, rural and green environments overlap. We find that the percentage of green space is indeed higher in natural environments (mean = 92%, median = 95%, s.d. = 10%, 5th percentile = 72%, 95th percentile = 98%) than built-up environments (mean = 58%, median = 62%, s.d. = 31%, 5th percentile = 9%, 95th percentile = 96%; $W=215\,930\,000$, $p < 0.001$, $N=119\,375$, Wilcoxon rank sum test with continuity correction), but that these two distributions clearly overlap; and that the percentage of green space is higher in rural environments (mean = 86%, median = 92%, s.d. = 16%, 5th percentile = 50%, 95th percentile = 97%) than outside rural environments (mean = 47%, median = 45%, s.d. = 27%, 5th percentile = 8%, 95th percentile = 89%; $W=4\,226\,400$, $p < 0.001$, $N=14\,228$, Wilcoxon rank sum test with continuity correction), but that again, there is clear overlap between the two distributions. We therefore find evidence that measurements of whether environments are natural, rural or green are highly related, but not exactly the same. Of most importance however, we find that scenic ratings are not entirely determined by any of these quantities. (Fig. 3a–c).

We investigate whether a relationship between scenicness and happiness is still found once these more traditional environmental measurements are included in the model. We determine the individual’s location at the time of polling, and consider whether the individual is located in a natural or built-up setting, an urban, suburban or rural environment, and what the percentage of green land cover is in the surrounding LSOA. As an additional check, we also include the log of median household income per LSOA^37^ as a control variable, as scenic areas may also be the areas in England in which inhabitants have higher incomes.

Again, Table 1 presents the results of our analysis. While our study accords with the hypotheses that people are happier in natural habitats ($\beta =0.574$, $CI=[0.303,0.844]$, $p < 0.001$, $N=138\,407$) and in rural locations ($\beta =0.608$, $CI=[0.263,0.954]$, $p < 0.001$, $N=138\,407$), we still find that participants report themselves to be happier when in more scenic areas ($\beta =2.770$, $CI=[1.757,3.783]$, $p < 0.001$, $N=138\,407$), even after controlling for this wide range of other characteristics of the local environment (Fig. 3d). We find the same results when we consider the environmental variables alone (Supplementary Table 3). Interestingly, our analysis does not provide strong evidence of an effect of green space on happiness ($\beta =-\,0.451$, $CI=[\,-\,0.999,0.0979]$, $p=0.107$, $N=138\,407$). Thus, we hypothesise the absence of the effect between green space and happiness could be because the variance of different wellbeing measures attributed to green space in previous studies has been captured by measures of whether the surrounding habitat is natural, rural or indeed scenic.

In these analyses, as noted in the *Methods* section, *Scenic-Or-Not* ratings have been rescaled from the original 1 (not scenic) to 10 (very scenic) scale rating to a 0–1 scale. Thus, an increase of 1 additional unit of scenicness in our analysis translates to an increase of 9 in the *Scenic-Or-Not* rating of a neighbourhood. On this basis, the predicted increase in happiness for each increase of 1 in the *Scenic-Or-Not* rating on the original rating scale is 0.308 on the 0–100 happiness scale. The predicted increase in happiness of someone moving from a neighbourhood with the lowest scenicness rating of 1 to a neighbourhood with a scenicness rating in the top 10% (i.e., a scenicness rating above 4.67), would therefore be 1.130 points on the 0–100 happiness scale. This is slightly below the increase in happiness observed when participants are sleeping, resting or relaxing (1.133), and greater than the increase in happiness observed when moving from a built-up environment to a natural environment (0.574) or when moving from a suburban environment to a rural environment (0.608). In the same fashion, the predicted increase in happiness of someone moving from a neighbourhood with the lowest possible scenicness rating of 1 to a neighbourhood with the highest possible scenicness rating of 10 would be 2.770 points on the 0–100 happiness scale. This effect is similar in size to the increase in happiness observed when participants are listening to music (2.336) and the decrease in happiness observed when participants are commuting (−2.214, Fig. 3d).

Finally, in order to explore whether data on scenicness can improve our understanding of environmental influences on happiness, given the explanatory power already offered by traditional environmental measurements, we compare three models. All three models contain the contextual control variables, such as weather, companionship and activities. The first model includes only data on scenicness. The second model includes data on scenicness, as well as the more traditional measurements of the local environment: whether *Mappiness* users were in a natural habitat, urban, suburban or rural environments; data on green space; and area-level median household income. The third model includes these traditional measurements yet excludes scenicness. In order to compare the fit of the models to each other, we calculate Akaike weights (AICw) following the method detailed in the *Methods* section. These weights can be interpreted as the probability of each model given the data, in the context of the set of possible models considered. Table 2 illustrates that there is very little evidence for a model which omits the data on scenicness. Instead, we find that the strongest evidence is found for the model including data on scenicness as well as traditional measurements of the characteristics of the environment.

**Table 2:** Comparing Models of the Influences of Location Characteristics on Happiness Ratings.

Models	AIC	AICd	AICw
With Scenicness only	1144476	51.8	<0.001
With Scenicness and Traditional Environmental Measurements	1144424.2	0	>0.999
With Traditional Environmental Measurements Only	1144465	40.8	<0.001

In order to further explore whether data on scenicness can help us understand changes in happiness, we compare three models. All three models contain the contextual control variables such as weather, companionship and activities. The first model includes only crowdsourced measurements of scenicness. The second model includes scenicness and the more traditional measurements of the local environment: whether *Mappiness* users were in natural, urban, suburban or rural environments, the percentage of green space in the local environment and the income of local inhabitants. The third model includes the traditional measurements of the local environment, yet excludes scenicness. To determine which model provides the best fit for predicting happiness, we calculate Akaike weights (AICw), which can be interpreted as the probability of each model given the data^47^. We find very little evidence for the model which does not include the data on scenicness. Instead, we find strongest evidence for the model that includes both the traditional environmental measurements and the crowdsourced measurements of scenicness.

### Scenic environments or taking a break

One further concern which could be raised about *in*-*situ* analyses of the relationship between characteristics of the environment and everyday happiness is that people may visit scenic or natural areas when they have the opportunity to take a break from their everyday routine. The *Mappiness* activity questions do allow us to measure whether individuals are undertaking activities that might be associated with holidays, such as sleeping, resting and relaxing, as well as whether an individual is at home, work or elsewhere, and we include these measurements in our fixed effects analysis. However, in order to verify that the holiday effect is not confounding our analysis, we also check whether the relationship between scenic environments and greater happiness still holds for individuals on weekends and bank holidays and when they are not at home or at work, when it could be argued that people might be more likely to be at leisure or taking a break from their daily routine. We find that the link between scenic areas and greater happiness is still robust ($\beta =4.261$, $CI=[2.550,5.972]$, $p < 0.001$, $N=35\,967$).

### Scenic environments and built-up settings

While there is limited scope to improve the beauty of natural settings, urban planners and policymakers do have the ability to influence the aesthetics of built-up areas^38^. We therefore split our data into data for built-up locations and for natural locations, and investigate whether the relationship between scenicness and happiness holds in both. Although the effect size is larger in natural settings, we find that within built-up locations too, people report themselves to be happier when in more scenic locations (natural: $\beta =5.756$, $CI=[3.249,8.262]$, $p < 0.001$, $N=37\,807$; built-up: $\beta =2.045$, $CI=[0.890,3.200]$, $p < 0.001$, $N=95\,113$; Fig. 3e).

## Discussion

Do individuals encountering more scenic environments during their everyday life experience greater levels of happiness? Here, we have presented what we believe to be the first large-scale study able to offer an answer to this question, through national scale measurements of the aesthetics of different environments and changes in happiness as thousands of individuals experience these various environments during their everyday life. We find that people are indeed happier in more scenic environments, even after controlling for a range of variables such as potential effects of the weather and the activity an individual was engaged in at the time. Crucially, we find that the effect of environmental aesthetics goes beyond the effect of whether an individual is in a natural, green or rural environment, and that even in built-up environments, people are still happier when the area they are in is more scenic.

We emphasise that built-up spaces can include natural elements such as grass (Fig. 2). A previous analysis of the *Scenic-Or-Not* dataset provided evidence that some natural elements such as forest paths, ponds and rivers do improve the scenic appeal of photographs taken in built-up areas^39^. However, the same analysis also showed that built elements such as churches, cottages and towers can positively impact scenic ratings too, and conversely, that some natural elements such as large quantities of grass can be associated with a negative impact on scenic ratings.

This distinction between aesthetic appeal and the presence of nature is vital if such research is to be used to inform policy decisions around the design and modification of built and natural environments. Our findings provide evidence that for built environments to be as conducive as possible to the wellbeing of their users, it may be wise for consideration to be given not only to whether areas of nature or green space have been included in the design, but to whether these natural areas are attractive – for which appropriate maintenance may well be required – and indeed to whether the buildings themselves are appealing to the eye. Similarly, our results provide evidence in line with the suggestion that if policymakers allow natural environments to be blighted by unsightly features, these environments may no longer provide the same wellbeing benefits for those who visit them.

Our results also have relevance for theories regarding the impact of our surroundings on our everyday happiness. The biophilia hypothesis suggests that we are likely to experience positive emotions in natural environments^7^. Our findings provide evidence that beauty may play an additional role in determining our emotions in a given environment, beyond the presence of nature. How might scenic settings therefore otherwise make us feel happier? According to Attention Restoration Theory^40^, scenes requiring less demand on our attention allow us to become less fatigued, more able to concentrate, and thus perhaps even less irritable. Such restorative settings have often been associated with nature, and in contrast, one can imagine that a bustling urban setting such as Times Square in New York might demand our full attention. However, more picturesque streets with broad views and fewer distractions might also function as restorative settings. Settings that are more beautiful may also hold our interest for longer, thereby blocking negative thoughts^9,41^. Furthermore, certain features of environments commonly associated with scenic environments, such as open spaces and spaces full of light, might make us feel happier simply because we feel safer^42,43^. This accords with prospect refuge theory^44^ as in such spaces one can easily observe “prospects” and avoid possible dangers. We do not rule out the possibility that characteristics of environments we consider “scenic” remind of us of environmental characteristics that we have found beneficial at some point in our evolutionary history. The connection we find between environmental aesthetics and everyday happiness may therefore still be due to evolutionary processes, as suggested by the biophilia hypothesis^7^, but not simply due to a preference for a connection with nature.

Our analysis does come with a number of limitations. A first limitation relates to how users of *Scenic-Or-Not* may have interpreted the core construct of ‘scenic’. We do not know how our results may have varied if the users had been asked to rate the photographs for ‘beauty’ or ‘attractiveness’. However, as previously noted, an earlier analysis of the *Scenic-Or-Not* data does provide some insight into the characteristics of an image that influence the ‘scenic’ measure^39^. It could also be argued that *Scenic-Or-Not* users might have considered a ‘scenic’ place to be a place in which they imagined they would feel happier. Even if this were the case however, our findings provide large-scale evidence that a separate group of people did then go on to experience more positive emotions when physically in these ‘scenic’ locations, and that this relationship was not simply explained by traditional measurements of the environment, such as whether it was natural or not, suggesting that the measure would still be a measure of value.

A second limitation is that our study focuses on a momentary measure of everyday happiness, rather than a broader analysis of subjective wellbeing. Subjective wellbeing is a multi-faceted notion composed of various elements^8^. This study therefore examines only part of the story regarding scenic environments and our subjective wellbeing, for example omitting potentially more stable long-term wellbeing measures, such as life-satisfaction.

A third limitation is that *Mappiness* users are all Apple iOS users. As Apple products are known for their design appeal, it might be that *Mappiness* participants are more likely to be affected by the aesthetics of their environment. A fourth concern might be that our scenicness ratings rely on individual photographs which might not be wholly representative of the aesthetics of the local area. Ratings of photographs might also be influenced by image composition or the weather depicted in the picture. However, despite these likely sources of noise, our analyses show that crowdsourced ratings of scenicness do help explain more variance in happiness than traditional environmental measurements alone.

Finally, and importantly, we acknowledge the limitations of our analyses in terms of the causal inferences that may be drawn. It is clear that while our findings offer evidence that more scenic locations are associated with more positive emotions, they do not conclusively demonstrate that scenic locations cause more positive emotions. In these analyses, we have taken a number of steps to help us progress towards a better understanding of whether or not such a causal relationship might exist. For example, we have attempted to account for a wide span of potential confounding factors, ranging from other characteristics of the environment, to various measurements of the situation in which an individual might find themselves when responding to the *Mappiness*. We have also used a modelling approach that helps us to capture other factors that might impact upon the happiness of an individual but do not change across time, such as personality traits. However, we fully recognise that other confounding factors may remain. Our analysis also does not rule out the possibility of reverse causality, such that people choose to go to more attractive locations when they feel happier.

Nonetheless, we have provided the first large-scale evidence of a relationship between beautiful environments and our everyday wellbeing. While current policy does suggest that policymakers see some value in the beauty of local environments, as demonstrated in the protection of Areas of Outstanding Natural Beauty and the development of attractive cultural quarters, the quality of such decisions is limited by lack of data on the beauty of environments^45^. For example, there is often very little guidance regarding the quality of green spaces, and thus more deprived areas might be prone to low-quality green spaces that have little appeal to local residents^46^. Our study takes an important step in providing evidence that the beauty of the environments we are exposed to in our everyday lives, and therefore decisions made in the design of such environments, might have consequences for people’s everyday happiness.

## Methods

### Scenic ratings

We measure scenicness using crowdsourced scenic ratings from *Scenic-Or-Not* (http://scenicornot.datasciencelab.co.uk/). *Scenic-Or-Not* presents users with random geotagged photographs, each representing one square kilometre of Great Britain, sourced from *Geograph* (http://www.geograph.org.uk). The *Geograph* web-based project aims to collect and reference geographically representative images of every square the British Isles. Photographers are required to photograph at close range one of the main geographical features within each square kilometre, and each photograph is then reviewed by a team of moderators (see https://www.geograph.org.uk/article/Geograph-or-supplemental for more details). The final *Scenic-Or-Not* database has over 217,000 images covering 92.5% of the 234,429 land mass 1 km grid squares of Great Britain. We use the mean rating of images that have been rated at least three times.

In order to ensure scenicness ratings are easily comparable to other dummy variables included in our analysis, we rescale the scenicness ratings to 0 to 1 prior to aggregating scenicness measurements on an LSOA basis. After scaling and aggregating scenic ratings per LSOA, the range of scenic ratings is 0.00 to 0.78. In other words, no LSOA has a perfect score of 1. For all of England, the region we use in our final analysis, we have 929,125 votes for 129,056 images, which gives us the ratings for 16,907 LSOAs out of the 32,482 LSOAs in England. Following combination with the *Mappiness* dataset as described below, our final scenicness ratings dataset contains 858,773 votes for 119,377 images, covering 14,228 LSOAs.

### Happiness ratings

To measure changes in happiness as individuals experience different environments, our study draws on happiness data from the Apple iOS smartphone app, *Mappiness*^3^. Prospective participants download the *Mappiness* app at no charge, indicate their informed consent to taking part, and provide basic demographic and health-related information. We consider a response to be valid only if the start time for the response is within 60 minutes of the most recent prompt by the iOS app, and the questionnaire is completed within 5 minutes. We only include responses that have a device-reported GPS accuracy of +/−250 m or better, and where the participant has reported that they are either “outdoors” or “in a vehicle”. We further exclude measurements collected in LSOAs where no *Scenic-Or-Not* image falls. The resulting dataset constitutes 138,407 measurements of momentary happiness, gathered from 15,444 users between June 2010 and June 2013, and covering 14,228 LSOAs out of the 32,482 LSOAs in England. We confirm that, other than where further exclusions are explicitly stated for particular sub-analyses in the manuscript, this is a full description of all data exclusions. The users report a median household income of approximately GBP 48,000, with a mean age of 35, and a female-to-male ratio of 48:52.

### Fixed effects model

Our basic fixed effects model for estimating happiness in more scenic environments is therefore as follows:

$${H}_{ilt}={\alpha }_{i}+{\beta ^{\prime} }_{s}{s}_{l}+{\beta ^{\prime} }_{p}{p}_{it}+{\beta ^{\prime} }_{r}{r}_{lt}+{\beta ^{\prime} }_{q}{q}_{l}+{\epsilon }_{ilt}$$

where *H*_*ilt*_ is an individual’s self-rated happiness, scaled from 0 (“not at all happy”) to 100 (“extremely happy”) at time *t* and location *l*; *α*_*i*_ is the unobserved individual-specific constant; *s*_*l*_ is the scenic rating of the LSOA *l*; *p* is a set of individual context control variables including companionship, activity; *r* is a set of time-variant weather control variables applying to a particular location, such as wind speed, cloud cover and temperature; and *q* is a set of environmental control variables that do not vary through time, such as percentage of green space, whether a setting is natural or built-up, whether an area is urban, suburban or rural, and the income of local inhabitants.

### Akaike weights (AICw)

In order to determine which model best captures variance in the data on happiness, we first calculate the AIC (Akaike Information Criterion) values for each model. AIC values help us to determine the likelihood of each model for a given set of data. The best model is the one that has the lowest AIC value. To help interpretation, we also calculate the Akaike weights of the models (AICws), following the method proposed by Wagenmakers and Farrell^47^. We derive AICws by first identifying the model with the lowest AIC. For each model, we then calculate an AIC difference, by determining the difference between the lowest AIC and the model’s AIC. We next determine the relative likelihood of each model, following the method described in Wagenmakers and Farrell^47^. To determine the AICws, we normalise these likelihoods, such that across all models they sum to one. The resulting AICws can be interpreted as the probability of each model given the data, given the full set of models considered in the analysis. The weights do not indicate the probability of the models in comparison to models that are not considered in the analysis.

### Weather data

Data on weather conditions have been taken from the Met Office Integrated Data Archive System (MIDAS) database^48,49^. In our analysis, we control for potential effects of wind speed (ranging from 0 to 44), cloud cover (ranging from 0 to 9), visibility (ranging from 0 to 7500), temperature (ranging from −18.70 to 30.60), sun (ranging from 0 to 1) and rain (ranging from 0 to 20) on happiness. All variables are scaled to take values in the range 0 to 1 prior to running the fixed effects analysis.

### Land cover data

To determine whether the environments that individuals experience are natural or built-up, we use data on land cover from the 25m-resolution UK Land Cover Map 2007 (LCM)^34^. Supplementary Table 1 lists which land cover types have been deemed as natural versus built-up.

### Green land cover data

Data on green space per LSOA have been taken from the Generalised Land Use Database Statistics for England 2005^36^.

### Urban, suburban and rural classifications

“Urban”, “suburban” and “rural” areas are defined using data from the 2011 Rural-Urban Classification^35^. We define “urban” LSOAs to be LSOAs in the category “Urban Major Conurbation”. LSOAs in the remaining urban categories in this classification are deemed “suburban”. In our final analysis, we consider data for the 3,226 urban LSOAs, 6,432 suburban LSOAs and 4,570 rural LSOAs for which we have scenicness and happiness data.

### LSOA level income data

As a metric of the economic environment an individual may be passing through at a given point in time, we consider the log of median household income of each LSOA, determined using Experian Demographic Data^37^.

## Supplementary information

Supplementary Information: Happiness Is Greater in More Scenic Locations

## Data Availability

This study was a re-analysis of existing data that are publicly available at locations cited in the reference section. Data on *Scenic-Or-Not* ratings are openly available at http://scenicornot.datasciencelab.co.uk. We retrieved scenicness ratings by accessing the *Scenic-Or-Not* website on 2nd August 2014. Data collected from the *Mappiness* app can be made available for reproducibility testing once appropriate agreements to ensure confidentiality and security are in place.

## References

[1] Bratman GN, Hamilton JP, Hahn KS, Daily GC, Gross JJ (2015). Nature experience reduces rumination and subgenual prefrontal cortex activation. P. Natl. Acad. Sci. USA.

[2] Hartig T, Evans GW, Jamner LD, Davis DS, Gärling T (2003). Tracking restoration in natural and urban field settings. J. Environ. Psychol..

[3] MacKerron G, Mourato S (2013). Happiness is greater in natural environments. Global Environ. Chang..

[4] van den Berg A, Maas J, Verheij R, Groenewegen P (2010). Green space as a buffer between stressful life events and health. Soc. Sci. Med..

[5] White M, Alcock I, Wheeler B, Depledge M (2013). Would you be happier living in a greener urban area? A fixed-effects analysis of panel data. Psychol. Sci..

[6] UK Parliamentary Office of Science and Technology (2016). Green Space and Health, POSTnote.

[7] Kellert, S. R. & Wilson, E. O. *The biophilia hypothesis* (Island Press, 1995).

[8] Diener E, Suh EM, Lucas RE, Smith HL (1999). Subjective well-being: Three decades of progress. Psychol. Bull..

[9] Ulrich RS (1979). Visual landscapes and psychological well-being. Landscape Res..

[10] Bakolis I (2018). Urban mind: Using smartphone technologies to investigate the impact of nature on mental well-being in real time. BioScience.

[11] Galindo MPG, Corraliza JA (2000). Environmental aesthetics and psychological wellbeing: relationships between preference judgements for urban landscapes and other relevant affective responses. Psychology in Spain.

[12] Pretty J, Peacock J, Sellens M, Griffin M (2005). The mental and physical health outcomes of green exercise. Int. J. Environ. Health Res..

[13] White M (2010). Blue space: The importance of water for preference, affect, and restorativeness ratings of natural and built scenes. J. Environ. Psychol..

[14] Lottrup L, Stigsdotter UK, Meilby H, Claudi AG (2015). The workplace window view: A determinant of office workers’ work ability and job satisfaction. Landscape Res..

[15] Rosenberg F (2009). The MoMA Alzheimer’s project: Programming and resources for making art accessible to people with Alzheimer’s disease and their caregivers. Arts & Health.

[16] Lazer D (2009). Computational social science. Science.

[17] Vespignani A (2009). Predicting the behavior of techno-social systems. Science.

[18] Hektner JM, Schmidt JA, Csikszentmihalyi M (2007). Experience sampling method: Measuring the quality of everyday life.

[19] Shiffman S, Stone AA, Hufford MR (2008). Ecological momentary assessment. Annu. Rev. Clin. Psycho..

[20] Kahneman D, Krueger AB, Schkade DA, Schwarz N, Stone AA (2004). A survey method for characterizing daily life experience: The day reconstruction method. Science.

[21] Redelmeier DA, Kahneman D (1996). Patients’ memories of painful medical treatments: real-time and retrospective evaluations of two minimally invasive procedures. Pain.

[22] Kahneman D, Thaler RH (2006). Anomalies: Utility maximization and experienced utility. J. Econ. Perspect..

[23] Doherty ST, Lemieux CJ, Canally C (2014). Tracking human activity and well-being in natural environments using wearable sensors and experience sampling. Soc. Sci. Med..

[24] Seresinhe CI, Preis T, Moat HS (2015). Quantifying the impact of scenic environments on health. Sci. Rep..

[25] Krekel C, Kolbe J, Wstemann H (2016). The greener, the happier? The effect of urban land use on residential well-being. Ecol. Econ..

[26] Stutzer A, Frey BS (2008). Stress that doesn’t pay: The commuting paradox. Scand. J. Econ..

[27] Ferrer-i Carbonell A, Gowdy JM (2007). Environmental degradation and happiness. Ecol. Econ..

[28] Richards J (2015). Don’t worry, be happy: Cross-sectional associations between physical activity and happiness in 15 European countries. BMC Public Health.

[29] Lelkes O (2006). Knowing what is good for you: Empirical analysis of personal preferences and the objective good. J. Socio. Econ..

[30] Rehdanz K, Maddison D (2005). Climate and happiness. Ecol. Econ..

[31] Kardan O (2015). Is the preference of natural versus man-made scenes driven by bottom–up processing of the visual features of nature?. Front. Psychol..

[32] Kotabe HP, Kardan O, Berman MG (2017). The nature-disorder paradox: A perceptual study on how nature is disorderly yet aesthetically preferred. J. Exp. Psychol. Gen..

[33] Ibarra FF (2017). Image feature types and their predictions of aesthetic preference and naturalness. Front. Psychol..

[34] Morton, D. *et al*. Land Cover Map 2007 (vector, GB) v1.2. NERC Environmental Information Data Centre (2014).

[35] Office for National Statistics (2013). The 2011 Rural-Urban Classification For Small Area Geographies.

[36] Department for Communities and Local Government (2007). Generalised Land Use Database Statistics for England 2005.

[37] Experian (2011). Household Income 2011.

[38] Reynolds, F. Urbanisation and why good planning matters. In *The fight for beauty* (Oneworld, London, 2016).

[39] Seresinhe CI, Preis T, Moat HS (2017). Using deep learning to quantify the beauty of outdoor places. R. Soc. Open Sci..

[40] Kaplan S (1995). The restorative benefits of nature: Toward an integrative framework. J. Environ. Psychol..

[41] Schertz KE (2018). A thought in the park: The influence of naturalness and low-level visual features on expressed thoughts. Cognition.

[42] Herzog TR, Chernick KK (2000). Tranquility and danger in urban and natural settings. J. Environ. Psychol..

[43] Loewen LJ, Steel GD, Suedfeld P (1993). Perceived safety from crime in the urban environment. J. Environ. Psychol..

[44] Appleton J (1996). The experience of landscape.

[45] Bakhshi H (2010). Beauty: value beyond measure.

[46] Roberts-Hughes R (2013). City Health Check: How design can save lives and money.

[47] Wagenmakers E-J, Farrell S (2004). AIC model selection using Akaike weights. Psychon. B. Rev.

[48] Met Office. MIDAS: UK Hourly Weather Observation Data (NCAS British Atmospheric Data Centre, 2006).

[49] Met Office. MIDAS: UK Hourly Rainfall Data (NCAS British Atmospheric Data Centre, 2006).

