# Supplementary material for: Happiness is Greater in More Scenic Locations
Source: Sci Rep. 2019 Mar 14;9:4498. doi: 10.1038/s41598-019-40854-6 (PMC6418136; doi:10.1038/s41598-019-40854-6)
Supplement: Supplementary file 1 — Supplementary Information: Happiness Is Greater in More Scenic Locations [file 41598_2019_40854_MOESM1_ESM.pdf]

Supplementary Information: Happiness Is Greater in More Scenic Locations

Chanuki Illushka Seresinhe,[1,2*] Tobias Preis,[1,2,3] George MacKerron[4]
and Helen Susannah Moat[1,2,3]

[1]Data Science Lab, Behavioural Science, Warwick Business School,
University of Warwick, Coventry, CV4 7AL, UK
[2]The Alan Turing Institute, British Library, 96 Euston Road, London, NW1 2DB, UK
[3]Department of Physics, Boston University,
590 Commonwealth Avenue, Boston, Massachusetts 02215, USA
[4]Department of Economics, University of Sussex,
Jubilee Building, Falmer, Brighton, BN1 9SL, UK

[*]To whom correspondence should be addressed; E-mail: C.Seresinhe@warwick.ac.uk.

Supplementary Note 1

Original URLs of scenic images in Fig. 2a, reproduced in full in Supplementary Fig. 3a, from top to bottom: Richard Swales (http://www.geograph.org.uk/photo/135301), Tony Atkin (http://www.geograph.org.uk/photo/189469), Tom Richardson (http://www.geograph.org.uk/photo/1012532), Helen Wilkinson (http://www.geograph.org.uk/photo/1108720); Original URLs of unscenic images in Fig. 2b, reproduced in full in Supplementary Fig. 3b, from top to bottom: Peter Whatley (http://www.geograph.org.uk/photo/986664), David Long (http://www.geograph.org.uk/photo/1000263), Mick Garratt (http://www.geograph.org.uk/photo/66880), Doug Lee (http://www.geograph.org.uk/photo/392588); Original URLs ofscenic built-up images in Fig. 2c, reproduced in full in Supplementary Fig. 3c, from top to bottom: Bob Jones (http://www.geograph.org.uk/photo/1154040), Phil D (http://www.geograph.org.uk/photo/1091209), Mike Searle (Fig. http://www.geograph.org.uk/photo/868144), Glyn (http://www.geograph.org.uk/photo/755931). Copyright of the images is retained by the photographers. Images are licensed for reuse under the Creative Commons Attribution-Share Alike 2.0 Generic License. To view a copy of this licence, visit http://creativecommons.org/licenses/by-sa/2.0/

Supplementary Figures

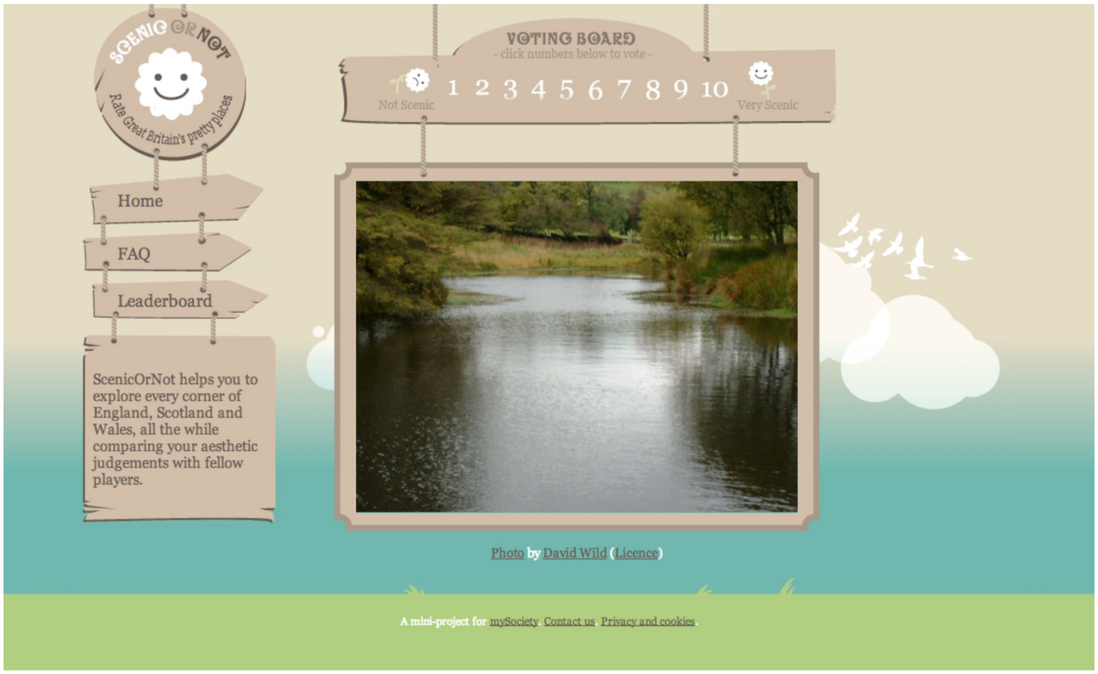

Supplementary Figure 1: The *Scenic-Or-Not* voting screen. *Scenic-Or-Not* presents users with random geotagged photographs of Great Britain, which they rate on an integer scale of 1 10 (10 indicating "very scenic" and 1 indicating "not scenic"). The *Scenic-Or-Not* database has over 217,000 images, sourced from *Geograph* (http://www.geograph.org.uk/), covering nearly covering 92.5% of the 234,429 land mass 1 km grid squares of Great Britain. *Scenic-Or-Not* was originally built by mySociety (https://www.mysociety.org/) and is now hosted by the Data Science Lab at Warwick Business School (http://scenicornot.datasciencelab.co.uk/). The data used in this study, including links to all images which were rated, can be downloaded at http://scenicornot.datasciencelab.co.uk/votes.tsv. Scenic image by David Wild (http://www.geograph.org.uk/photo/35940). Image is licensed for reuse under the Creative Commons Attribution-Share Alike 2.0 Generic License. To view a copy of this licence, visit http://creativecommons.org/licenses/by-sa/2.0/.

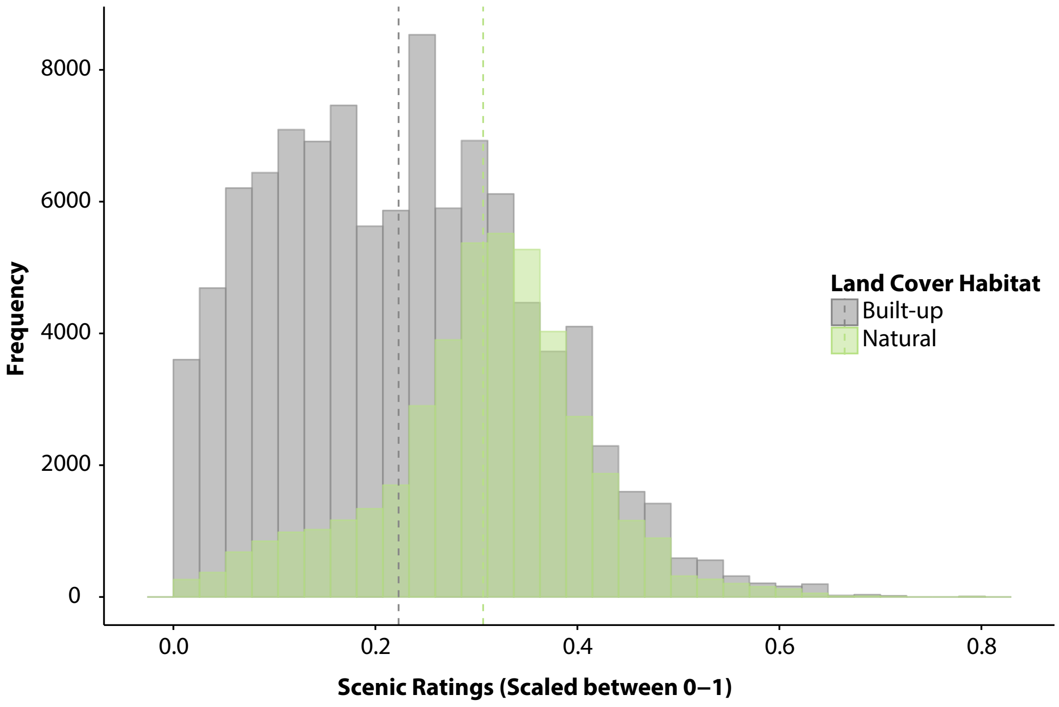

Supplementary Figure 2: Scenicness in built-up versus natural locations. We use land cover type data from the 25m-resolution UK Land Cover Map 2007[1] and categorise each location for which we have a rating of scenicness as either a natural or a built-up environment. Table 1 lists land cover types deemed as natural and those deemed as built-up. Scenic ratings tend to be higher in natural environments (marked green) than in built-up environments (marked grey). However, the distributions of ratings exhibit considerable overlap.

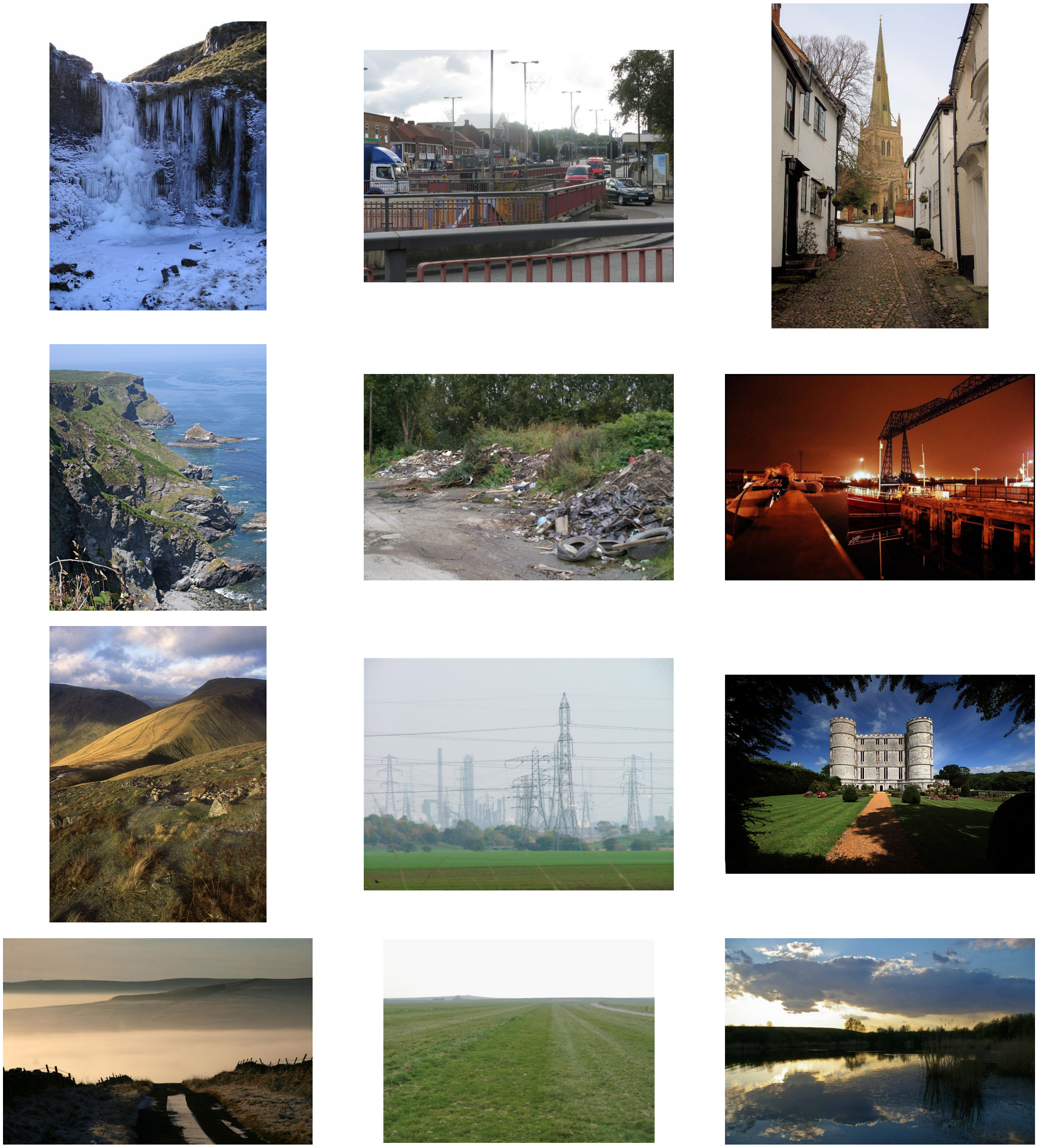

(a) (b) (c)

Supplementary Figure 3: Full images from Fig. 1. See Supplementary Note 1 for credit, copyright and license details.

Supplementary Tables

Supplementary Table 1: Land Cover Data.

LCM2007 class	Habitat
Broadleaved woodland	Natural
Coniferous woodland	
Arable and Horticulture	
Improved Grassland	
Rough Grassland	
Neutral Grassland	
Calcareous Grassland	
Acid Grassland	
Fen, Marsh and Swamp	
Heather	
Heather grassland	
Bog	
Montane habitats	
Inland Rock	
Salt water	
Freshwater	
Supra-littoral Rock	
Supra-littoral Sediment	
Littoral Rock	
Littoral Sediment	
Saltmarsh	
Urban (including Bare and Urban)	Built-up
Suburban (including Urban industrial and Urban suburban)	

In order to determine whether an environment is natural or built-up, we use data on land cover type from the 25m-resolution UK Land Cover Map 2007 (LCM)[1] to categorise each location for which we have scenic ratings as either a natural environment or a built-up environment. The table shows which land cover types have been deemed as natural, and which have been deemed as built-up.

Supplementary Table 2: *Mappiness* activities.

Working, studying	Texting, email, social media
In a meeting, seminar, class	Browsing the Internet
Travelling, commuting	Watching TV, film
Cooking, preparing food	Listening to music
Housework, chores, DIY	Listening to speech/podcast
Admin, finances, organising	Reading
Shopping, errands	Theatre, dance, concert
Waiting, queueing	Exhibition, museum, library
Childcare, playing with children	Match, sporting event
Pet care, playing with pets	Walking, hiking
Care or help for adults	Sports, running, exercise
Sleeping, resting, relaxing	Gardening, allotment
Sick in bed	Birdwatching, nature watching
Meditating, religious activities	Hunting, fishing
Washing, dressing, grooming	Computer games, iPhone games
Intimacy, making love	Other games, puzzles
Talking, chatting, socialising	Gambling, betting
Eating, snacking	Hobbies, arts, crafts
Drinking tea/coffee	Singing, performing
Drinking alcohol	Something else (version $<$ 1.0.2)

The table above lists all the *Mappiness* activities that respondents can choose. All the activities were included as control variables in our analysis.

Supplementary Table 3: Estimated Model Parameters For Fixed Effects Model without *Mappiness* Predictor Variables

	Model 1: scenicness only		Model 2: scenicness and environmental variables	
Environment Variables	*Coeff*	*95% C.I.*	*Coeff*	*95% C.I.*
Scenicness	10.39***	[9.062,11.72]	6.705***	[5.372,8.038]
Natural habitat	—	—	2.231***	[1.922,2.540]
Percentage of green space	—	—	-0.0550	[-0.757,0.647]
Log of area-level median household income	—	—	-0.178	[-0.697,0.340]
Urban	—	—	-1.256***	[-1.749,-0.764]
Rural	—	—	0.807***	[0.380,1.235]
Suburban (base category)	—	—	—	—
Observations	138,407		138,407	
Groups (participants)	15,444		15,444	
Groups (LSOAs)	14,228		14,228	
R^2	43.2%		43.4%	
Adjusted R^2	36.0%		36.3%	
Within R^2	0.5%		1.0%	

* $p < .05$, ** $p < .01$, *** $p < .001$

Supplementary References

[1] Morton, D. *et al.* Land Cover Map 2007 (vector, GB) v1.2. NERC Environmental Information Data Centre. (2014).
